# Supplementary figures and images for: Evaluation of serum MMP-2 and MMP-3, synovial fluid IL-8, MCP-1, and KC concentrations as biomarkers of stifle osteoarthritis associated with naturally occurring cranial cruciate ligament rupture in dogs
Source: PLoS One. 2020 Nov 19;15(11):e0242614. doi: 10.1371/journal.pone.0242614 (PMC7676649; doi:10.1371/journal.pone.0242614)

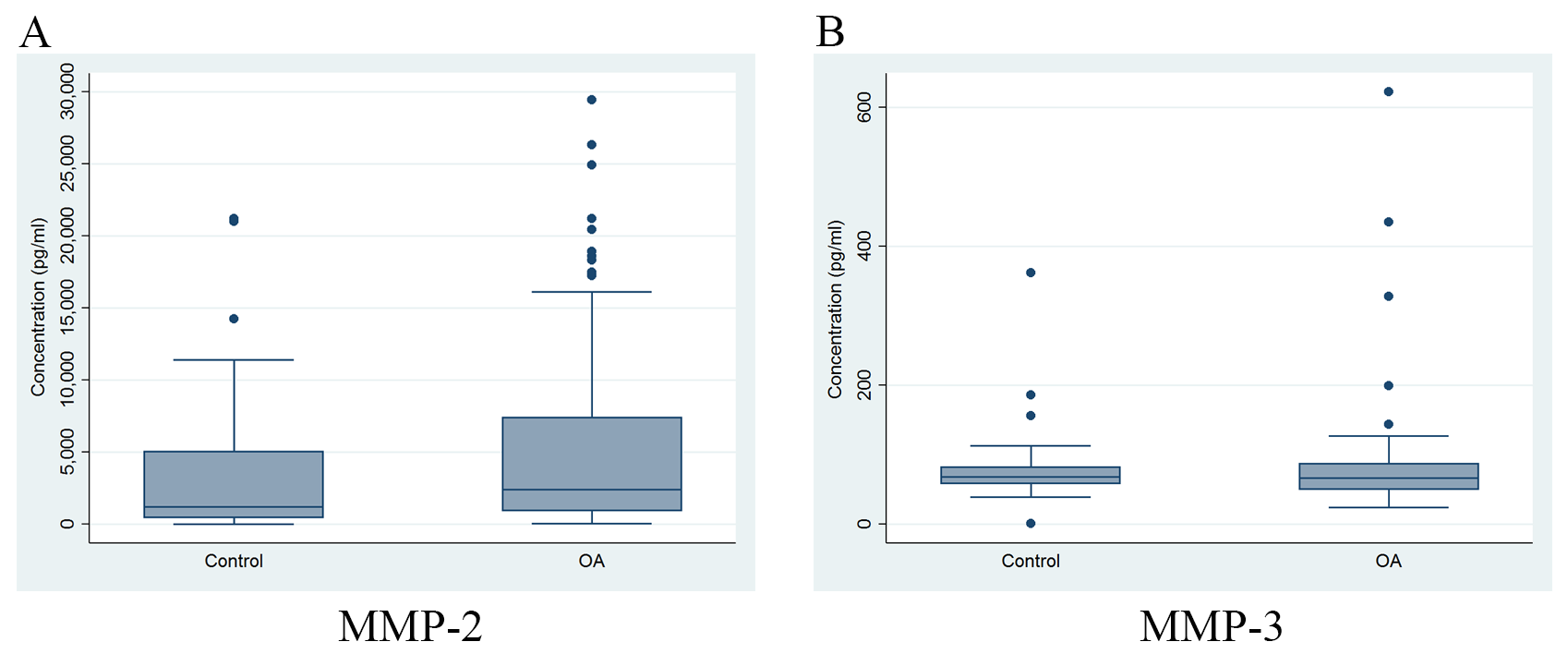

Supplement: S1 Fig — Distribution of MMP-2 (A) and MMP-3 (B) serum concentration for control and osteoarthritis (OA) groups (pg/ml). The horizontal line inside each box is the median and the upper and lower edges of box present the inter-quartile range (IQR). The whiskers are either 1.5 × IQR or the range, whichever is smaller. Dots outside the fences are outliers. (TIF) [file pone.0242614.s001.tif]

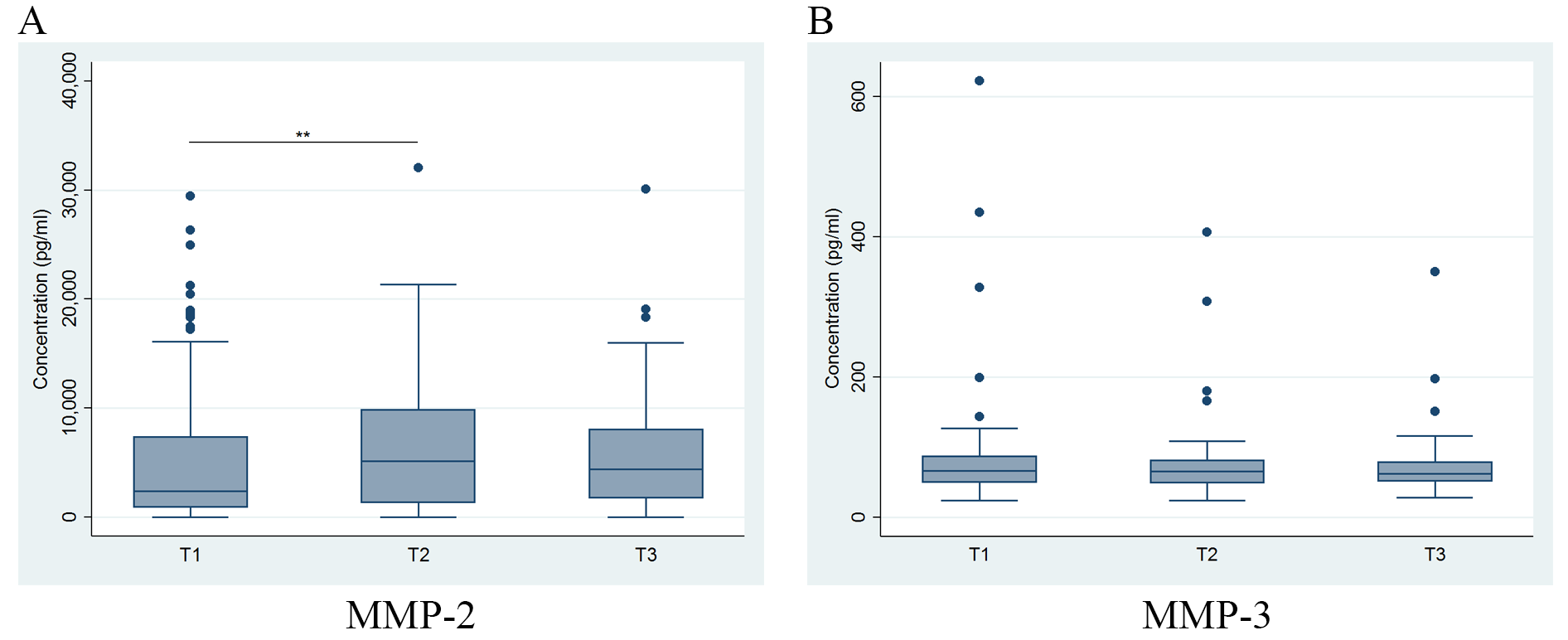

Supplement: S2 Fig — Distribution of serum concentrations of MMP-2 (A) and MMP-3 (B) over three time points for the OA group dogs. T1: initial visit, T2: 4-week recheck, T3: 12 week recheck. The horizontal line inside each box is the median and the upper and lower edges of box present the inter-quartile range (IQR). The whiskers are either 1.5 × IQR or the range, whichever is smaller. Dots outside the fences are outliers. ** Statistically significant different concentrations (P < 0.01). (TIF) [file pone.0242614.s002.tif]
