# Supplementary material for: Evaluation of serum MMP-2 and MMP-3, synovial fluid IL-8, MCP-1, and KC concentrations as biomarkers of stifle osteoarthritis associated with naturally occurring cranial cruciate ligament rupture in dogs
Source: PLoS One. 2020 Nov 19;15(11):e0242614. doi: 10.1371/journal.pone.0242614 (PMC7676649; doi:10.1371/journal.pone.0242614)
Supplement: Attachment — Submitted filename: PlosOne R2 Response to Reviewers.docx [file pone.0242614.s005.docx]

PONE-D-20-13103R1

Evaluation of serum MMP-2 and MMP-3, synovial fluid IL-8, MCP-1, and KC concentrations as biomarkers of stifle osteoarthritis associated with naturally occurring cranial cruciate ligament rupture in dogs. PLOS ONE

Dear Dr. Zhang and our reviewers,

We would like to thank you all for your constructive feedback and recommendations. We hope that this version meets your expectations and that you find our responses to your comments appropriate.

**Response to Academic Editor**

We did not see any specific comments from you regarding the manuscript’s content itself. However, we are looking into seeing if the protocols.io applies to us and will be getting our protocol added there. We have included the tracked and untracked version of the manuscript along with all the supplementary files and figures. Our financial disclosure and ethics statement has not changed. It appears that our reviewers did not have significant concerns during this review; therefore, our response to their comments is limited to the only point raised in section “6. Review comments to the author”. We also followed the recommended PACE software to check the figure requirements, and Figure 1 was the only file that was adjusted by the system. We uploaded the updated version in the new submission. We also added the data spreadsheet in a compressed format as a supplementary file to fulfill the data availability requirement of the journal.

**Response to reviewers’ comments:**

We have revised the check box to allow data availability in response to reviewer 3’s answer.

Reviewer #3: The authors have thoroughly considered the many comments from the reviewers, and in my opinion made appropriate adjustments and corrections. The nature of the study makes it exploratory - all overstatements have been reformulated. Also excess figure/tables have been moved to supplementary, giving the manuscript a better flow.

Authors’ response: We very much appreciate your feedback and are happy that our efforts have met your expectations.

Authors’ action: None.

Reviewer #4: I believe that the authors have addressed the limitations previously highlighted by reviewers. I have noted that the authors took into account the recommendations to be more cautious in describing the markers they are highlighting as having a discriminatory value rather than using diagnostic, particularly in the discussion. I therefore would like to recommend that they use this terminology consistently when describing their objectives and throughout the paper.

Authors’ response: Thank you very much for your positive feedback. We agree that the diagnostic phrase can be misleading, as we did not evaluate these biomarkers in differentiating this form of OA from other arthritic conditions. We appreciate you noticing this issue throughout the paper.

Authors’ action: We have removed the term “diagnostic” when referring to the current biomarkers’ performance and ability and have simply used the term discriminative performance/ability throughout the paper. We have highlighted areas where the term “diagnostic” was removed. We chose the term discriminative over discriminatory to be more particular in the use of the word as an adjective.
